# Supplementary material for: Good practices to optimise the performance of maternal and neonatal quality improvement teams: Results from a longitudinal qualitative evaluation in South Africa, before, and during COVID-19
Source: PLoS One. 2024 Nov 19;19(11):e0314024. doi: 10.1371/journal.pone.0314024 (PMC11575831; doi:10.1371/journal.pone.0314024)
Supplement: S1 Table — (DOCX) [file pone.0314024.s001.docx]

**S1 Table: Mphatlalatsane partnerships**

| **Partner** | **Role and responsibilities** |
| --- | --- |
| National Department of Health | Mphatlalatsane custodian; provides strategic focus |
| Clinton Access Health Initiative (CHAI) | Implementation secretariat; coordinate and direct day-to-day activities; provides technical support through QI advisors |
| South African Medical Research Council/University of Pretoria Maternal and Infant Health Care Strategies Research Unit (SAMRC-UP) | Content expert providing input and support for maternal healthcare strengthening activities |
| University of Limpopo Trust Initiative for Newborn Care (ULT) | Content expert providing input and support for neonatal healthcare strengthening activities |
| Institute for Healthcare Improvement (IHI) | Technical support to advisors, QI training (September 2019 - June 2020) |
| South African Medical Research Council (SAMRC) and University of the Western Cape (UWC) | Evaluation team |
