# Supplementary material for: Good practices to optimise the performance of maternal and neonatal quality improvement teams: Results from a longitudinal qualitative evaluation in South Africa, before, and during COVID-19
Source: PLoS One. 2024 Nov 19;19(11):e0314024. doi: 10.1371/journal.pone.0314024 (PMC11575831; doi:10.1371/journal.pone.0314024)
Supplement: S3 Table — (DOCX) [file pone.0314024.s003.docx]

**S3 Table: Services delivered at participating health facilities**

| **Facility type** | **Services** |
| --- | --- |
| **Clinic** | It provides routine and preventive healthcare services, such as vaccinations, antenatal and postnatal care, screening and treatment for communicable and non-communicable diseases [1]. |
| **Community healthcare centre** | The centre provides similar services to a primary healthcare clinic, with the addition of a 24-hour maternity service, emergency care and casualty and a short-stay ward. The CHC will refer a patient to a district hospital when necessary [2]. |
| **District hospital** | The hospital receives support from general specialists based at regional hospitals. It may only provide the following specialist services: (a) paediatric health services; (b) obstetrics and gynaecology; (c) internal medicine; (d) general surgery; and (e) family physician [3]. |
| **Tertiary hospital** | The hospital offers specialist level healthcare services [1]. |

**References**

1. Kwa Zulu Natal, Department of Health. (n.d). [accessed 21 June 2024]; Available from: <https://www.kznhealth.gov.za/definitions.htm>.
2. Phungula, N., L. Robertson, and S. Mall, Views held by South African primary health care nurses on caring for people living with mental illness. South African Journal of Psychiatry, 2024. 30(1): p. 2148.
3. Matolengwe, A., D. Murray, and U.B. Okafor, The Challenges of Implementing a Health Referral System in South Africa: A Qualitative Study. Risk Management and Healthcare Policy, 2024. 17(null): p. 855-864.
