# Supplementary material for: Good practices to optimise the performance of maternal and neonatal quality improvement teams: Results from a longitudinal qualitative evaluation in South Africa, before, and during COVID-19
Source: PLoS One. 2024 Nov 19;19(11):e0314024. doi: 10.1371/journal.pone.0314024 (PMC11575831; doi:10.1371/journal.pone.0314024)
Supplement: S4 Table — (DOCX) [file pone.0314024.s004.docx]

**S4 Table: Consolidated Framework for Implementation Research (CFIR) - Domain 3**

**Inner setting comprises:**

QI team, hosting

facility, and other QI teams in the same facility and / or district

**CFIR domain 3: Inner setting**

**Research questions**

**Data sources**

**Participants**

1. Advisors
2. Team members
3. Team leaders

**Methods**

1. Programme documentation review
2. Interviews / FGDs
3. Advisor debriefing

**Interview guide: key questions**

1. The ‘what’, ‘how’, and ‘when’ of the team’s interaction with other programmes in the facility, and other teams
2. What are the facility-level barriers and enablers of team performance and team leadership?
3. What role does the facility manager play regarding team performance and team leadership?
4. Does the team have a pre -Mphatlalatsane history of working together and does it appear to influence current team performance and leadership?
5. How functional is the facility in aspects other than Mphatlalatsane activities, and does this appear to be related to team performance and team leadership?
6. What are the perceptions of how the team functions?

- interaction with other facility staff and programmes
- interaction with other QI teams in the same and different facilities respectively

1. What do team members and team leader respectively, perceive as facility-level barriers and enablers regarding their team - and leadership performance?

6. What are the team leaders’ and team members’ perceptions and experiences of the impact of COVID-19 on their QI activities and the facility services?

On
