# Supplementary material for: Good practices to optimise the performance of maternal and neonatal quality improvement teams: Results from a longitudinal qualitative evaluation in South Africa, before, and during COVID-19
Source: PLoS One. 2024 Nov 19;19(11):e0314024. doi: 10.1371/journal.pone.0314024 (PMC11575831; doi:10.1371/journal.pone.0314024)
Supplement: S5 Table — (DOCX) [file pone.0314024.s005.docx]

**S5 Table: Interview foci**

| **Leaders and members** | **Advisors** |
| --- | --- |
| - Training - How teams were established - Operationalising their quality improvement work - Experiences of being a leader / member - Team performance, successes, and challenges - Enablers and barriers at facility and district level - COVID-19 impact on service delivery and team functioning | - Their activities and engagements with teams - Assessments of leader and team performance - Enablers and barriers of quality improvement uptake, including the how district support shaped uptake |
