# Supplementary material for: Good practices to optimise the performance of maternal and neonatal quality improvement teams: Results from a longitudinal qualitative evaluation in South Africa, before, and during COVID-19
Source: PLoS One. 2024 Nov 19;19(11):e0314024. doi: 10.1371/journal.pone.0314024 (PMC11575831; doi:10.1371/journal.pone.0314024)
Supplement: S6 Table — (DOCX) [file pone.0314024.s006.docx]

**S6 Table: Assessment evidence per facility**

*Well-performing teams*

| **District** | **Facility code** | **Team performance** | **Evidence** |
| --- | --- | --- | --- |
| 1 | Facility 1 | Well | - Evidence of team structure and functional team   “So, they’ve got a team leader and then they’ve got a scriber. So, the scriber, you get every information that you need.” (adv, doc18)   - Leader QI enthusiast and positive about team performance   “So, I’m so much in love with it.” (leader, doc63)  “We are just a team. We also have a WhatsApp group called Z, saying we are smart as obstetrics team” (leader, doc62)   - Evidence of sustaining QI activities during the COVID-19 period of August 2020 - March 2021: continued implementation of change idea - Team matured |
|  | Facility 4 | Well | - Evidence of team structure and functional team: active leader with strong support from OPM   “WO: Would it be fair to say that they are the core people of Mphatlalatsane?  R: Yes.” (leader, doc130)  “if I’m not on duty, sister A has to check the files.” (leader, doc130)   - Leader QI enthusiast and positive about team performance   “WO: Ja. Are you happy with how you performed with the QI work?  R: Ja, it has worked for us a lot. It has worked for us a lot, especially the completion of those records.” (leader, doc130)  “R: The other people who are working together at the maternity section, they are all advanced midwifery. Yes, so when you are doing all these things, all of us, we are hands-on when we are doing our project” (leader, doc130)   - Evidence of sustaining QI activities during the COVID-19 period of August 2020 - March 2021: continued with implementation of change idea and initiated 1 new change idea - Team matured |
|  | Facility 5 | Well | - Evidence of team structure and functional team: during Baseline interview leader had the three core team members present and explained each one’s responsibility.   “I allocated her to manage maternity, so I thought it was good of me to take her with me so that she can spearhead this project.” (leader, doc58)   - Leader QI enthusiast and positive about team performance   “I found Mphatlalatsane as enriching. It’s enriching my mind. It’s enriching my staff. It’s not an extra load.” (leader, doc58)  “R: There was no problem at all. They didn’t see it as additional work.  WO: And what was the secret to that success?  R: Working together. Yes, involving them in what you’re doing and telling them and giving them feedback on the targets.” (leader, doc127)   - Evidence of sustaining QI activities during the COVID-19 period of August 2020 - March 2021: continued with implementation of change idea and initiated 1 new change idea - Team matured |
|  | Fac 6 | Well | - Evidence of team structure and functional team   “R: So, I was the one who was running this project since the beginning of the project until the end. But we work as a team. I don’t work alone. Yes, everybody is involved.” (leader, doc126)   - Leader QI enthusiast and positive about team performance   “So, what motivates you when you say it brings more work, but yet you do the more work? What’s the benefit for the facility in that?  R: So, we are now relearning quality service to our patients because we have to look at each and everything that you are dealing with for the patient.” (leader, doc126)  “Or let me rather ask, do you think that you are a good team at the moment?  R: Yes, because everyone is involved. Everyone knows how to go about it. Everyone knows how to deal with these challenges.” (leader, doc126)   - Evidence of sustaining QI activities during the COVID-19 period of August 2020 - March 2021: continued with implementation of change idea and initiated 3 new change ideas - Team matured |
| 2 | Facility 3 | Well | - Evidence of team structure and functional team   “b: Yes, I gave the report to all the staff members. So mostly I took the staff from the maternity because they are the ones who are hands-on on seeing the antenatal clients.” (leader, doc49)   - Leader QI enthusiast and positive about team performance   “b: Yes, you’re right, because after we’ve seen that we have achieved in QIP 1, we immediately started on QIP 2.” (leader, doc50)  “No, the team still works well.” (leader, doc123)  “are you happy with your maternity team’s performance?  R: Ja.” (leader, doc123)   - Evidence of sustaining QI activities during the COVID-19 period of August 2020 - March 2021: continued with implementation of change idea and initiated 1 new change idea - Team matured |
|  | Facility 5 | Well | - Evidence of team structure and functional team   “It’s me, being the overseer, a is the QIP champion, and then Sister B being the QI deputy champion.” (leader, doc60)   - Leader QI enthusiast and positive about team performance   “I can say we bought it because our passion is for us to improve.” (leader, doc59)   - Evidence of sustaining QI activities during the COVID-19 period of August 2020 - March 2021: continued with implementation of change idea and initiated 1 new change idea - Team matured |

*Less well-performing teams*

| **District** | **Facility code** | **Team performance** | **Evidence** |
| --- | --- | --- | --- |
| 1 | Facility 2 | Less well | - Evidence of team structure but not functional team after leader left in 2022; during End line interview with members, it was not clear who is taking responsibility for QI activities. - Leader QI enthusiast but struggled to get staff to participate.   “WO: But the use of PDSA was striking. Is it jargon that you picked up from Mphatlalatsane? Or was it specific to the…  b: No, it came actually from looking online for how to improve things in a way that’s proven to be successful and one of the tools that came up quite frequently was the PDSA cycle.” (leader, doc47)  “For example, for me, ja, I find it very useful as an individual, but then when I come back and try and explain to others, we didn’t speak the same language and then it was quite difficult to motivate.” (leader, doc47)   - No evidence of sustaining QI activities during the COVID-19 period of August 2020 - March 2021 - Team not matured |
|  | Facility 3 | Less well | - Evidence of team structure but not functional team; had 2 leaders during implementation.   “Sr C who was leader for most of ’20-’21 told me she retires end Jun22. The OPM who was leader after training but on sick leave end ’19 / early ‘22 returned around Aug21” (149)   - Leader not QI enthusiast and struggled to get staff to participate.   “the leader was saying she is running in circles and not winning with any of the c/ideas. No buy-in from doctors” (fieldwork journal, doc149)  “So, I’m still on the gaps that we identified in 2019, even now. I don’t see me getting one of it resolved” (leader, doc117)   - No evidence of sustaining QI activities during the COVID-19 period of August 2020 - March 2021 - Team not matured |
| 2 | Facility 1a | Less well | - No evidence of team structure and disbanded when Covid started. - There was only an End line interview jointly with the 3 sisters from the original 2 teams.   “three people in the labour ward that are incapacitated.  R: It’s two, sister a …  WO: Okay, now I’m with you.  R: The one that is incapacitated is Sister D. Sister E was off sick. She wasn’t well for a period of time” (adv, doc16)   - Leader not QI enthusiast and complained about staff shortage.   “Dr A who was nominated as a QI champion was moved to another facility in District 2, Sr F is a QI trained clinician has been medically boarded. There has been changes in leadership, the CEO at Facility A is acting.” (adv, doc142)  “So, for the neonatal unit, Sister G, who had also attended, is sort of the core lead or the team lead for the neonatal unit” (adv, doc14)  “WO: Sister, I assume that you are the leader at this point in time. Or am I wrong?  R: I was just supporting Dr A project leader” (leader, doc116)   - No evidence of sustaining QI activities during the COVID-19 period of August 2020 - March 2021 - Teams not matured |
|  | Facility 1b | Less well |  |
|  | Facility 2 | Less well | - No evidence of team structure and functional team   “The ward sister even less enthusiastic than Facility Z, and also not clear on who the M’tsane champions are / Only worthwhile thing will be to figure out why it is like this” (fieldwork journal, doc148)   - Leader not positive about team performance   “We don’t work as a team.” (leader, doc42)  “Sometimes you want to call them to give the feedback, so you’ll find that they are too busy” (leader, doc120)   - No evidence of sustaining QI activities during the COVID-19 period of August 2020 - March 2021 - Team not matured |
|  | Facility 4 | Less well | - No evidence of team structure and functional team   “Not clear how much of M’tsane is still intact” (fieldwork journal, doc148)  “And what are they working on and how is Sister H’s nightshift affecting work?  R: It has affected the work because she was a great acting manager and now there are two managers that are acting that we met with when we were there at the facility, sister a as well as sister b” (adv, doc16)   - Leader not QI enthusiast and not positive about team   “I don’t know what they are expecting from me to do. Because I’m just trying to do the things that they just ** when they need statistics, what to do and how to make the graphs to see how we are performing in our facility.” (leader, doc26)  “R: For the team, I can say the first time it was high. Then it was abandoned on the way because our team leader just went out of the project. Because she was our acting manager and then she was out. Then you’ll find that there are gaps. We continued with the project, but we didn’t have a leader and we didn’t meet." (leader, doc114)   - No evidence of sustaining QI activities during the COVID-19 period of August 2020 - March 2021 - Team not matured |
| 3 | Facility 1 | Less well | - No evidence of team structure and functional team: when the new District 3-4 advisor came, the team was so dysfunctional that her 1^st^ priority was to do teambuilding with them. - Leader neutral about QI and negative about team performance   “So, each and every person is busy in their corner, and we can hardly meet.” (leader, doc39)  “but the challenge is us meeting on a month-to-month basis, on a quarterly basis because everyone is overwhelmed. There’s too much work. This one is doing that and that one is doing that.” (leader, doc38)  “But at the same time, we have ideas that we bring up, how do I make sure that these change ideas we initiated and said we were going to do, have been done. That’s a difficult one because all these departments have operational managers who need to see to that.” (leader, doc118)   - No evidence of sustaining QI activities during the COVID-19 period of August 2020 - March 2021 - Team not matured |
| 4 | Facility 2 | Less well | - No evidence of team structure and functional team: there were member names on paper, but: “The other people were merely onlookers, if I can put it like that.” (leader, doc29)      - Leader not QI enthusiast and negative about team performance   “if I ignore it, it will go away and I won’t have to look at it, that was the approach we had.” (leader, doc29)  “We are clinicians, we’re not researchers. We don’t know how to do research.” (leader, doc29) “   - No evidence of sustaining QI activities during the COVID-19 period of August 2020 - March 2021 - Team not matured |
|  | Facility 3 | Less well | - Evidence of team structure and functional team:   “I involved only myself, the other PN [professional nurse] I had at that time, the enrolled nurse next-door because she’s the one doing most of the pregnancy testing when they come in, and my outreach team.” (leader, doc55)   - Leader QI enthusiast and staff implemented QIP, but she never felt it necessary that they be interviewed as she carried all the QI responsibilities. Yet their QIPs were effective.   “All these different things, these fishbones and all of these things that I’ve never used before. So ja, that was quite exciting.  WO: I can see that it made sense to you.  R: It made sense to me. It’s still making sense to me. I’m still using all those methods to solve little things in the facility.” (leader, doc55)  The advisor observed tension between the leader and her staff members:  “Because she has clinicians who have left the facility because they were not getting along. Sister I, the clinician who is there now, she is not the first one. She also wants to go. So, they need to go to the root of the problem instead of changing the clinicians because they don’t get along.” (adv, doc109)   - No evidence of sustaining QI activities during the COVID-19 period of August 2020 - March 2021 - Team not matured |
