# Supplementary material for: Good practices to optimise the performance of maternal and neonatal quality improvement teams: Results from a longitudinal qualitative evaluation in South Africa, before, and during COVID-19
Source: PLoS One. 2024 Nov 19;19(11):e0314024. doi: 10.1371/journal.pone.0314024 (PMC11575831; doi:10.1371/journal.pone.0314024)
Supplement: S7 Table — (DOCX) [file pone.0314024.s007.docx]

**S7 Table: Indexing and charting Mphatlalatsane CFIR constructs**

| **Domain and associated constructs** | **Definition** | **Mphatlalatsane operationalisation** |
| --- | --- | --- |
| ***Domain 1: Intervention characteristics*** | Intervention description | Plan-Do-Study-Act model |
| Adaptability | Degree to which intervention components can be adapted:   - core - without these it is not the intended intervention - peripheral - can be adapted without compromising the intervention | - Core elements: data - driven process, testing, audit tools, evidence - Peripheral elements: rapid cycles, weekly meetings, prescribed stationery |
| Complexity | Perceived difficulty reflected by duration, disruptiveness, and number of steps | - The duration and number of steps of the change idea |
|  | | |
| ***Domain 3: Inner setting*** | Organisation in which the intervention is implemented | - Existing facility service team/s - QI team |
| Culture | - Organisation’s norms and values | - Work culture in existing service team/s |
| Compatibility | - Integrate intervention with existing systems | - Embedding quality improvement activities in routine practices |
| Learning climate | - Leader acknowledges her/his fallibility - Members feel safe to own the intervention | - Leader models ‘To err is human’ - Leader creates psychological safety for members |
| Resources | Resources available | Existing staff and small budget requests |
|  | | |
| ***Domain 4: Individuals*** | Individuals who implement the intervention | Leader, Members, Facility management, Advisors |
| Knowledge and beliefs about intervention | Attitude towards the intervention and enthusiastic implementation | Leaders’ enthusiasm |
| Other attributes | Other characteristics that are important for intervention implementation | Leader’s tenacity |
|  | | |
| ***Domain 5: Implementation process*** | Intervention implementation processes |  |
| Engaging | Involve appropriate individuals | - Establish core team with second in-charge leader - Facility management support |
| External change agents | External individuals assist with intervention implementation | Advisors |

*Definitions and abbreviations*

Audit tool: An existing / self-initiated document to record change idea implementation and effectiveness

Data driven process: Use data to identify service delivery problems and assess change idea effectiveness

Existing service teams: Staff providing maternal and neonatal services at the facility

Prescribed stationery: Standard PDSA stationery
