# Supplementary material for: Good practices to optimise the performance of maternal and neonatal quality improvement teams: Results from a longitudinal qualitative evaluation in South Africa, before, and during COVID-19
Source: PLoS One. 2024 Nov 19;19(11):e0314024. doi: 10.1371/journal.pone.0314024 (PMC11575831; doi:10.1371/journal.pone.0314024)
Supplement: S8 Table — (DOCX) [file pone.0314024.s008.docx]

**S8 Table: Maternal and neonatal health issues addressed through change ideas**

| **Facility type** | **Health issue** |
| --- | --- |
| **Clinics** | Anaemia in pregnancy  Antenatal first visit before 20 weeks (x2 facilities)  Antenatal TB screening  Antenatal viral load monitoring  Pregnancy induced hypertension |
| **Community healthcare centres** | Anaemia in pregnancy  Antenatal first visit before 20 weeks (x3 facilities)  Antenatal TB screening  Antenatal viral load monitoring  Improving recording HIV testing at birth  Integration of lay health workers in maternal health referrals  Long-acting reversible contraception uptake rate  Maternal electronic gatekeeping (eGK) codes for HIV viral load monitoring  Partogram completion  Teenage pregnancy |
| **District hospitals** | 3rd stage labour to reduce post-partum haemorrhage  Antenatal first visit before 20 weeks  Asphyxia  Completion of partogram (x2 facilities)  Hypoglycemia in newborn babies in the neonatal unit  Pre-eclampsia |
| **Tertiary hospitals** | Monitoring fetal movements during pregnancy  Children under 1 year immunised  Neonatal infection prevention  Nosocomial infection in the neonatal unit  Triage pregnant women on arrival  Viral load completion during labour |
