# Supplementary material for: Good practices to optimise the performance of maternal and neonatal quality improvement teams: Results from a longitudinal qualitative evaluation in South Africa, before, and during COVID-19
Source: PLoS One. 2024 Nov 19;19(11):e0314024. doi: 10.1371/journal.pone.0314024 (PMC11575831; doi:10.1371/journal.pone.0314024)
Supplement: Attachment — Submitted filename: Response to Reviewers_15Oct24.pdf [file pone.0314024.s010.pdf]

Response to reviewer comments
(15/10/24)

Submission ID
PONE-D-23-40800R1

Title
Good practices to optimise the performance of maternal and neonatal quality improvement teams: results from a longitudinal qualitative evaluation in South Africa, before, and during COVID-19

Dear Reviewer
We truly appreciate your time for such a detailed review and providing us with text and suggestions to improve the manuscript. Kindly find our responses in the table below. Please note that the line numbering in the table refers to the Tracked changes version of the manuscript,

Kindly note that following another proofread of the text, we made the following self-initiated changes:
1. We made two changes to Fig 3 to highlight two good practices that were missing in the previous version; we attached a version in which we detailed the two changes.
2. We corrected grammatical errors in lines 98, 301, and 538.
3. We changed "service delivery problems" to "service delivery challenges" as the latter has a more positive tone; see the changes in lines 424, 541, 600, 645, and 683.

Response to reviewer table

**Reviewer comment**	**Authors' response**
1/<br>-Bottom of page 5 "*The evaluation and evaluation team were independent…*" reads a bit oddly. Perhaps just "The evaluation team was…"	Lines 110-3: Done.
2/<br>-Very minor, but for the numbered list on page six, please replace the commas at the end of 1 and 2 with semi-colons	Lines 117-8: Done.

Reviewer comment	Authors' response
3/<br>-I'm a bit surprised (given that this was supported by IHI) that it wasn't the collaborative model for QI with learning sessions being indicated. Not necessarily an issue, just different language than what IHI usually adopts.	Lines 149-51: We detailed why the Mphatlalatsane QI model was not a fully-fledged IHI collaborative model.
4/<br>-You refer to advisors and note "henceforth 'advisors'" on pg. 5, so you don't need to repeat on pg. 8.	Line 153: Corrected, thanks for picking it up.
5/<br>-On page 8, please refer to "districts" when you're referring to more than one (e.g. "districts 3 and 4" vs "District 3 and 4").	Corrected throughout: lines 154, 157, 159, 160, 190, 198.
6/<br>a. -It's a bit confusing on pg. 9 that you note only one facility declined due to COVID interruptions, but then note another team in District 4 declined (and there is one team per facility).<br><br>b. To this sentence please revise to "…their own participation was sufficient" to add clarity. That being said, in line 168 you note 15 teams included from the consenting facilities—but if there were only 14 consenting facilities (with 15 teams), and if one team declined, then should this be 14? You might want to then clarify that there were 14 facilities,<br><br>c. 17 leaders (1 per facility plus replacements due to turnover) and 14 teams (14/15 facility teams, one facility with two teams) that participated. Otherwise, please clarify	a. Lines 169-72: We moved the lines about the 15 teams to immediately follow the sentence of 14 consenting teams.<br><br>b. Lines 172-3: clarified that a District 4 leader agreed to her being interviewed but declined the participation of her team.<br><br>c. We left the 17 leaders across the 15 teams as there were 2 replacement leaders in line 183, but repeated in line 183 that there were 14 participating facilities.

**Reviewer comment**	**Authors' response**
7/<br>-Please swap the semi-colons in line 175 with commas.	Now line 185: Done.
8/<br>-In line 187, please change "initially" to "initial" (or change to "three initially-appointed…") and add "being" before "replaced"	Now line197: Done.
9/<br>-Please remove the comma from line 209.	Now line 231: Done.
10/<br>-In line 219 the hyphen is not necessary.	Now line 241: Hyphen deleted.
11/<br>-Please add a colon after "focused on" so that the list is introduced.	Line 252: Done.

Reviewer comment	Authors' response
12/<br>a. - Please provide a bit more information about how the interview guides were developed/what they included.<br><br>b. Further, (if you can) please make the links between data collection and analysis. For instance, as you analysed against CFIR (which I imagine was quite the undertaking…), I would have expected to see CFIR domains being covered in data collection to facilitate this, but the information that's present suggests that was not the case. Otherwise it's rather like shoehorning your data into a framework that may not actually work. Basically, there may be a disconnect between data collection and analysis. If so, consider adding this as a limitation to your discussion.	 a. (i) Lines 258-61: We added that the interview guides were informed by the CFIR and QI literature.<br>(ii) We think that 'interview guide' reads better that 'interview schedule' and changed throughout.<br><br>b. (i) We used the CFIR to inform our data collection, and moved the CFIR text under Analysis to Data collection, lines 214-226. We provided examples (lines 219-21) and Supporting information S4 table: CFIR - Domain 3, of how the framework was used.<br>(ii) Other members of the evaluation team evaluated Domain 2 of the framework, and we added text to explain this, see lines 225-7 and an earlier reference to this, lines 110-1.
13/<br>-Please introduce what you are listing (presumably the steps by Goldsmith et al) from line 286.	Please see if the changes, lines 302-5 clarifies this.
14/<br>-Please define how you are using "life course" within the confines of this research as it's not quite described.	Please see if lines 322-4 clarify this sufficiently.

Reviewer comment	Authors' response
15/<br>a. -You note that you use a framework analysis, but your results for sections 2 and 3 (though both indeed appearing to be a framework analysis) were derived differently, for instance, with CFIR only being used in section 2 (if it was used for section 3, that is not evident and would need explaining) versus being more thematic in section 3 with the inductively derived themes (the bolded "subsections" in the results, to me, are your themes and I would call them as such). It's not quite clear how you arrived at your analysis for section 3.<br><br>b. Also related to your analysis/results, I really like the "good practice #..." at the end of each results section, but it needs to be clearer from your analysis how you arrived at these. Can you please revise so that wording of the analytical process/results better align?	a. We added clarifying text, lines 327-32 and 354-5, to clarify that the 'indexing and charting' resulted in Section 2, and through the comparative thematic analysis we mapped and interpreted the data. This allowed us to develop the life stages as themes in Section 3.<br><br>b. We added text to clarify, lines 330-2.
16/<br>-Further to this, please add a line to explain why you chose to structure your analysis around CFIR for section 2 versus the many other analytical approaches that could have been taken.	Our aim, as outlined in the Abstract and Introduction, was to understand the contextual factors and implementation processes that facilitate or hinder implementation. This guided our framework choice, leading us to select the CFIR framework because it explicates possible contexts / processes that may shape implementation. Kindly see the text we added:<br>• Abstract, lines 52-3, and also see lines 293-7.
17/<br>a. -It's not clear how the team performance assessment analysis was taken into consideration in the later analysis. Please make this more explicit.	a. We added the following text, in tracked changes below (line 331)<br>"We then carefully reviewed the salient implementation processes per life stage for the well-performing teams, and from these, identified the 'good practices'."

**Reviewer comment**	**Authors' response**
b. Critically, it's also not quite clear how you populated the team performance assessment—<br>(i) what exactly were those preliminary sources of data?<br><br>(ii) And how did you ensure this wasn't a totally subjective exercise?	b(i). For each criterion, the method is listed (lines 279-85)<br>• *Leadership:* … as observed during data collection<br>• *Reviving QI:* Evidence, from reviewing programme documentation …<br>• *Leader attitude:* … reported during leader - and member interviews and advisor debriefings<br>• *QI maturity:* … reported in the interviews and debriefings<br><br>b(ii). We believe the data presented in S6 Table: Assessment evidence per facility counters by and large researcher bias. For convenience sake, here is an example (S6 Table, District 1, Facility 5, Well performing team, p1-2)<br><br>*Leadership*<br>• Evidence of team structure and functional team: during Baseline interview leader had the three core team members present and explained each one's responsibility.<br><br>*Reviving Q*<br>• Continued with implementation of change idea and initiated 1 new change idea<br><br>*Leader attitude*<br>• "I found Mphatlalatsane as enriching. It's enriching my mind. It's enriching my staff. It's not an extra load." (leader, doc58)<br>• "There was no problem at all. They didn't see it as additional work." (leader, doc58)<br><br>*QI maturity*<br>• Team matured

**Reviewer comment**	**Authors' response**
18/<br>-Please rename "Flowchart 1" to "Figure 1" and swap with Table 6 so that the table comes before the figure as the table facilitates figure interpretation.	Done, but it is actually Figure 2, as Figure 1 is the map. Previous Fig 2 (life course graphic) is changed to Fig 3, line 405.
19/<br>-Please add a legend for Figure 2 (e.g. define HCWs, QI, and QIP)	Added, lines 406-9.
20/<br>-In line 440, please remove the "a" before "curiosity"	Now line 459: Done.
21/<br>-In line 447 add "use" (or something to that effect) after "data"	Now line 466: Changed to 'aptitude to use routine data'.
22/-For good practice 2, I wonder if it's more "..and train them to tailor their support to facilitate team independence…" or something along those lines?	Very good suggestion, see lines 521-2.
23/<br>-Please add a comma after "respectively" in line 500 and remove the comma from 501.	Now lines 530-1: Done.
24/<br>-In 514 please replace "it" with "them"	Now line 541: Done.
25/<br>-Please remove the underscore from line 525	Now line 555: Done, and thanks for spotting it.
26/<br>-Please change "team" to "teams" in line 526	Now line 556: Done.

Reviewer comment	Authors' response
27/<br>-Please clarify lines 533/34. It's not clear what "it" in line 534 is referring to.	Now lines 56-63: Please see if the change clarifies this.
28/<br>-It's not clear for me how good practice 4 reflects the preceding results. Consider expanding these slightly to make the link clear.	Lines 571-2: We added "positive work culture".
29/<br>-Please add "both" before "less" in line 542	Now line 575: Done.
30/<br>-Good practice 5: Standards of care usually refer to clinical standards relating to treatment, etc. I think you may mean "standard practice". Please reflect across Figure 2 and also in line 684.	Now lines 593 and 719: Done. We changed this also in lines 526 and 575, and in the flowchart (Fig 2), we changed 'routine practice' to 'standard practice'. We also corrected S6 Table accordingly.
31/<br>a. -Please change the comma in line 565 with a colon<br>b. and add a colon after "included" in line 593.<br>c. In general, whenever you have items in a list separated by semi-colons, they need to be introduced with a colon.	a. Now line 600: Done.<br>b. Now line 628: Done<br>c. Thanks, we corrected it throughout the manuscript, lines 89, 104-5, 175, 676, and 732.
32/<br>-In line 565/66, I think the steps should be plural (e.g. "the change idea" should be "change ideas").	Now line 600: Done.
33/<br>-In line 574/583/695 please write "2nd" as "second" and "1st" as "first" in line 603	Now lines 609, 613, 618, and 730: Done. We also corrected this in the flowchart and S6 Table.

**Reviewer comment**	**Authors' response**
34/<br>-Please remove the hyphen from 575 and the comma in line 596.	Now lines 610, 631: Done.
35/<br>a. -Sorry for the semantics, but in 589, the change idea wouldn't (or shouldn't) be "partogram completeness" but what they proposed to put in place to support more completion of partograms.<br>b. Likewise, in 604/5, reducing PPH, partogram completion, and reducing neonatal sepsis aren't change ideas but aims. Please revise.	a. Now line 624: Now reading as: "the change idea was to improve partogram completeness."<br>b. Now lines 640-2: We clarified that the change ideas aimed to improve postpartum haemorrhage ….
36/<br>-In line 606 you refer to the S7 file as a list of change ideas, but these are just problems to which change ideas were applied. Please revise.	Now lines 641-2: Changed to "List of health issues that the Mphatlalatsane change ideas targeted."
37/<br>-Please de-capitalise "triage" in line 613	Now line 649: Done.
38/<br>-For all indented and italicised quotations, please remove the quotation marks around them.	Done.
39/<br>-Please write it as "QI-specific" in line 640	Now line 675: Done. We also changed it throughout the text for consistency.
40/<br>-Please add a colon after "as" in line 641	Now line 676: Done.
41/<br>-Please change "leader" to "leadership" in line 646	Now line 681: Done.

Reviewer comment	Authors’ response
42/<br>-Please remove the comma from line 651	Now line 686: Done.
43/<br>-Please replace “should thirdly,” with “should, therefore, thirdly,” in line 659	Now line 694: Done.
44/<br>-Please add a comma after “documented” in line 667 and delete the second one in line 669	Now lines 702 and 704: Done.
45/<br>-Throughout, where you refer to “contexts” I think “contextual factors” (e.g. line 702) reads more clearly.	Now line 736: Done. We also amended lines 49, 118, 294, 669, 736, 739, and 811. We checked for the same issue in the Supporting information tables.
46/<br>-You refer to “prevailing culture in existing service teams” as being a “lesser reported” issue, but I’d argue there’s a substantive literature about this, although it’s often referred to as a “culture of QI” or “improvement” or “teamworking” (juxtaposed to a “culture of blame”), which I would recommend you cite here.	We agree, and thanks for pointing it out. We removed ‘lesser reported’, lines 739-40, and referred back to the learning climate, lines 750-1.
47/<br>-Please replace “timely” with something like, “within good time” in line 719	Now line 755-6: Done.
48/<br>-In line 720, should “strengthen” be “strengthened”?	Indeed, now line 756, done.
49/<br>-In line 724 please revise to “…well, but are likely…”	Now line 760-1: Done, reads now: “may have played a role in why some teams performed less well.”

**Reviewer comment**	**Authors' response**
50/<br>-Change "infer" to "infers" in line 725	Now line 761: Done.
51/<br>-Consider deleting 728–735. I think it's clear that the good practices reflect what was needed in less well-performing teams and present in the well-performing ones.	Agree, now lines 764-71 removed.
52/<br>-Lines 737–739 are confusing. Throughout the manuscript, manuals are not raised, nor is implementation research. I would delete this after the [60,80] reference and then move to the comment about longitudinal data.	Agree, and changed accordingly, now lines 774-5.
53/<br>-You don't refer to stationery (line 751) elsewhere. Please remove.	Now line 787: Done.
54/<br>-You note the value of the program management committee meetings, but it's not evident how insights from these meetings are reflected in your results. Please incorporate within your results.	We added an example of what we learned about the change in the scope of the advisors' work, lines 490-7, added a quote, lines 499-502, and added text, lines 790-1, that refer back to the example.
55/<br>-Please delete the hyphen in lines 762 and 765	Now line 799, 803: Done. We also amended throughout the manuscript.

**Reviewer comment**	**Authors' response**
56/<br>a. -In your limitations, please acknowledge that the leader will also likely not speak freely about the "performance" of the members/the team.<br>b. Further, the leader who declined their team's participation—this is interesting and I wonder if it's less to do with their time and more to do with not wanting them to have the opportunity to speak.	It is a good point, and we added in lines 800-01.<br><br>This may very well be the case, but I had no interactions at all with the members to confirm or deny this assumption. The only evidence I have on why the leader did not want them to participate, is the reason she offered at recruitment was the one stated in lines 172-3.
57/<br>-As Reviewer 2 noted, please remove the spaces, throughout the manuscript, around hyphens.	Done.
