# Supplementary material for: Good practices to optimise the performance of maternal and neonatal quality improvement teams: Results from a longitudinal qualitative evaluation in South Africa, before, and during COVID-19
Source: PLoS One. 2024 Nov 19;19(11):e0314024. doi: 10.1371/journal.pone.0314024 (PMC11575831; doi:10.1371/journal.pone.0314024)
Supplement: Attachment — Submitted filename: Response to Reviewers_04Aug24.docx [file pone.0314024.s009.docx]

**Response to reviewer comments**

(04/08/24)

**Submission ID**

PONE-D-23-40800

**Title**

Good practices to optimise the performance of maternal and neonatal quality improvement teams: results from a longitudinal qualitative evaluation in South Africa, before, and during COVID-19

*Dear Reviewers*

Thank you for taking the time to review our manuscript. We are grateful for your considerate and constructive suggestions that improved the quality of the manuscript.

Kindly note the following self-initiated changes:

1. Edited the *Introduction section* to shorten the manuscript.
2. We shortened the text throughout the manuscript. In many instances it is only deleting a word in a sentence, for example: “Though some ~~of the~~ domains …”. In other instances where the shortened text is more substantial, it is highlighted with the following comment: “Self-initiated changes in this section are to shorten the manuscript.”.
3. Collated the previous Tables 2 and 3 (leader and member demographics respectively) into one, now Table 2, (p11) to shorten the paper.
4. Revised Table 1, p7, by adding the total number of Mphatlalatsane facilities to make it clearer that we selected 14 of the 21 ‘Mphatlalatsane facilities’ for the evaluation.
5. We added text of the ethical approval we received from the University of the Western Cape and the research committees from the departments of health in the respective provinces, p19, lines 318-25.

**Reviewer 1**

| **Editorial comment** | **Authors’ response** |
| --- | --- |
| Thank you for submitting a high quality article, tackling an important, relevant and current issue, namely, quality improvement in maternal and neonatal health services. Please check and make the below suggested minor corrections: | Thank you. |
| 1/Abstract: Please revise the statement in lines 39-41 for better readability/understanding: "To identify good practices to improve quality improvement teams’ performance, by identifying optimal facility-level contexts and implementation processes." | Corrected, now p3, line 47. |
| 2/  Findings: The findings are insightful. However, as the study is framed from the outset as a longitudinal qualitative evaluation, perhaps it might be helpful to go beyond a description of the time sequence in the background/ introduction/data collection sections to bring out this longitudinal element in the findings as well. It is great that the time sequence for recruitment, trainings, implementation is brought out in detail but then how QI processes/outcomes changed (or did not change) with time isn't very clear in the findings, dimming the study's distinctive longitudinal aspect. To clarify, are any of the good practices or contextual factors shaped by time lapse? | We did not find changes in team performance / adaptations over time; teams rather got set in how they used / not used QI; please see p41, lines 787-92. |

**Reviewer 2**

| **Editorial comment** | **Authors’ response** |
| --- | --- |
| 1/   a. This manuscript describes an important project with key lessons about improving maternal and neonatal healthcare services in diverse organizations in low and middle-income countries, Overall, the work merits publication. b. However, the manuscript raised several questions, and the language wasn't always entirely clear. | a. Thank you. b. We did a thorough language edit. |
| 2/ In the Introduction, it isn't clear if the first sentence applies to all countries or to low-and-middle income countries only. | Please see the clarification, p4, lines 64. |
| 3/  The definition offered of healthcare quality is limited. Perhaps the definition from the Institute of Medicine (US) Committee on Quality of Health Care in America in the publication, Crossing the Quality Chasm: A New Health System for the 21st Century, would better suit the purpose of this work because the IoM definition specifically includes consideration of implementation of effective evidence-based practice. | Thanks for the useful resource which we added to the definition, p4, line 68. |
| 4/  In line 60, the word models is used twice. | With shortening the text this was removed. |
| 5/  Reference is made to the Consolidated Framework for Implementation Research as the basis of this evaluation. The version cited of the CFIR is 2009. If the updated version from 2022 had been used, would it have made any difference to the evaluation and the findings? | In all likelihood it would have, as it recommends deciding a priori on the research outcomes. However, as detailed on p18, lines 297-9, our fieldwork was almost done and we already commenced with the analysis when this was published, and therefore unable to account for it in the study. |
| 6/  Under Setting, the distribution of facilities involved in the work seems appropriate for the delivery of the services in South Africa. However, it would be helpful to know a little more about the institutional maternal mortality ratio, for example, is it applied nationally and what does it mean, and if performance on this ratio was a factor in the selection of the participant facilities. | The evaluation team was not involved in selecting the facilities, nor privy to the data on which the National Department of Health selected the facilities (p6, lines 114-6). We added a description of the process, as shared by the Mphatlalatsane Programme management committee, p7, lines 134-8. |
| 7/  On page 7, it is unclear what a "partner" is and how they were identified, designated or selected. | We clarified that NDoH invited them, p9, lines 164-6, and their roles are described in S1 Table: Mphatlalatsane partnerships. |
| 8/  On page 8, under Leaders, line 164 refers to both midwives and nurses. This is a little confusing. Is the experience cited all in midwifery, or in the nursing profession, which may not have been specializing in midwifery. | We changed ‘in nursing’ to ‘as health professionals’, p9, lines 184-5, and also Table 2, p11-2. |
| 9/  On page 10, under Advisors, it wasn't clear why there were so many resignations from this role. Did the resignations have anything to do with the advisory role and barriers or difficulties faced by the advisors who resigned? | As evaluation team we were not informed about the reasons for the resignation, and did not have contact with the advisors post-resignation nor permission to add this question to our interview guide; see clarifying text, p13, lines 205-9. |
| 10/  Under Team Performance Assessment, page 13, beginning with line 242:   a. What was the basis for the criteria selected to define well-performing teams, and b. how were decisions made that teams met or didn't meet the criteria, that is, the operational definitions of the criteria. | a. We did not set the criteria when drafting the protocol. It originated during data collection and the early stages of analyses, with the data beginning to show how teams differed. The four criteria were the most obvious markers separating the teams who were actively doing QI from those who did not. We added text that explains this (p17, lines 267-76). b. The evidence on which we based the assessment is provided in S5 Table: We added more detail on the ‘Evidence criterion’ in the table (p1-3) for each of the well-performing facilities. |
| 11/  Page 16 and following, section 2, see previous comment on the version of the CFIR used for this work, and if the updated version would have changed the approach to analysis. It was useful to see how the CFIR was used, but the question is just about the version of CFIR and would it have made any difference if the current version of the CFIR had been used. | Please see our response to Comment 5. |
| 12/   a. Page 25, Intervention compatibility with standard care. The point made earlier about the definition of quality is relevant to the concept of implementing "standard care". The text in this section made an extremely critical point, and the good practice point derived from the text is very valuable for many teams trying to achieve substantial improvements in their services. b. Overall, the good practice points drawn out in the manuscript are helpful practical points for others. c. The content on Implementation processes and Context also is very helpful for those who might be taking on a similar project in similar circumstances. d. The study strengths and limitations were worth noting. | Thank you. |
| 13/  An overall concern about the manuscript are the slips in English grammar. A few examples:   a. In the Abstract, the second statement (lines 39 to 41) is not a complete sentence. b. A consistent issue is the lack of subject and verb agreement, for example, page 4, line 84, the verb should be is not are; c. line 89, the verb should be has not have. d. Also, commas were inserted when they were not appropriate. | Thanks for pointing this out; please see our response to Comment 1b.   a. Corrected, now p3, line 47. b. With shortening the text this was removed. c. Corrected, now p5, line 96. d. Corrected throughout the text. |
| 14/  Overall, the manuscript might be stronger if it were shorter. The authors could consider if some of the content could be summarized or shortened. | We made the following changes to shorten the paper:   - Kept the CFIR domain definitions and how these were operationalised in Table 6 (p24), and moved the detailed of construct definitions and its operationalisation to S6 table. - Collated previous Tables 2 and 3, participant demographics, into 1, now Table 2, p11-2. - Deleted text throughout the paper, e.g., In some instances we just deleted a word, but for more substantial shortening, we added the following comment: “Self-initiated changes in this section are to shorten the manuscript.”. |

**Reviewer 3**

| **Editorial comment** | **Authors’ response** |
| --- | --- |
| This paper discusses its findings into how to produce effective QI within healthcare using the Plan-Do-Study-Act (PDSA) method. They discuss the characteristics of what makes a good QI leader, advisors, and the need for supporting team members. | n/a |
| Strengths: - The paper explores the users’ perceptions on what worked and didn’t work when implementing QI practices into maternal and neonatal healthcare services. - The paper provides some interesting ideas that need further exploration - For the most part, the paper is well written and easy to follow However, there are some areas that if improved upon would make for a better submission: | Thank you. |
| 1/  Abstract - The abstract does not clearly detail what this study is about, does not clearly identify the need of this study, and provides unnecessary detail in the abstract (i.e., analysed using Altas.ti). | We clarified the text, please see Abstract, p3, line 47, and deleted ‘Atlas.ti’. |
| 2/  Abstract: Additionally, it didn’t clearly define what the core contribution of this work is. It appears as though the contribution is: How to establish effective QI teams within healthcare settings to improve health outcomes: a case study. | We added text to clarify; please see Abstract, lines 52-4; p.6, lines 118-22. |
| 3/  Introduction:   a. The paper starts off by discussing the use of PDSA in the context of maternal and neonatal healthcare services. However, the body of the results focus on generalised outcomes that the authors already recognise as previously discussed, as most of the discussion was stating that they ‘confirm’ what others have already established. b. I was expecting the paper to more heavily focussed on how PDSA was used to identify how to improve maternal and neonatal healthcare, c. what the main obstacles are towards implementation within the context of this work, and d. suggestions about how this could be improved in future studies. | a. It is unclear to us what the reviewer means with: “the body of the results focus on generalised outcomes”? If this refers to the Discussion section, we contend that the nuances of our results are novel and describe them in detail in our response to Comment 10. b. We understand. In the paper, we are trying to balance the need to provide key theoretical insights with attention to the specificities of the PDSA model. With respect to the latter, kindly see:  - p32, lines 586-90 on who developed and implemented the PDSAs; and - added: - an example of a successful change idea, p33, lines 613-7; - details about the maternal and neonatal topics commonly addressed in the PDSAs, p34, lines 624-8; - a table (S7) that details the topics per facility type; - (p38, lines 717-8) the reference to the paper in which we described in detail how the PDSA was used, and adapted (Odendaal et al., 2023: <https://doi.org/10.1186/s12913-023-09826-5>); and - also see p30, lines 529-34 on how the PDSA model was adapted.  c. We detailed staff shortages and keeping night shift members, p26, lines 437-8; added the high-pressure environment of delivery in hospitals, p29, lines 518-22; and lack of management support, p35, lines 643-4. d. We see this as addressed in the good practices distilled from the data analysis. |
| 4/  Methodology   a. - It isn’t clear as to why PDSA was used rather than six-sigma or lean, b. but I also expected to have a more indepth discussion about the use of PDSA in the methodology and how it was used in the context of this study. | a. As the Evaluation team we had no say in the National Department of Health’s choice of QI model, and added text clarify this, see p8, lines 149-50. We also clarified that we had no say in any of the Mphatlalatsane’s implementation decisions, p6, lines 114-6. b. Kindly refer to our response to Comment 3b. We also added S3 Table with information on the types of services offered by the different facility types. |
| 5/  Methodology - What type of interviews were conducted? Where they semi-structured or structured and why? | Semi-structured; added in Table 3 (p13), and added the rationale, p16, lines 246-8. |
| 6/  Methodology   a. - What is framework analysis, b. why did you use this method? Being that you had interview data, why not use deductive thematic analysis? | a. We added text to explain this, p17, lines 285-9. b. We chose the Consolidated Framework for Implementation as the study’s theoretical framework as we found many examples of it being used for evaluating QI programmes. The framework is useful as it identifies the implementation and contextual factors that shaped team performance in this study. |
| 7/  Results - Table 6 and the paragraph below it give the exact same information, please decide which method you think best portrays this information. | We deleted the text (p20, lines 342-4), and kept Table 5, p20. |
| 8/  Results  - You use pronouns when discussing your participants instead of anonymising them with they/them/their | We changed ‘he / she’ to ‘they/them/their’ throughout the text, and added text to explain that it was done to further anonymise participants, p19, lines 330-2. |
| 9/  Results  - I found that most of the supporting qualitative data was from the well-performing teams. It would have been interesting to delve deeper into what impacted some teams more than others and how could these be better alleviated? | Our data does not allow for this level of analysis and added it is a limitation, p42, lines 816-8. |
| 10/  Results  - Your good practices boxes were interesting, but seemed very ‘well known’ for the most part. | - To our knowledge, Mphatlalatsane is the second MNH QI programme in SA reported in peer-reviewed literature that focus on health services beyond those specifically for mothers and babies living with HIV. We regard it important that this study confirmed global QI evidence on the importance of process, context, leader skills, and teamwork, in the context of maternal and child health, in a high HIV prevalence, LMIC setting and during an ongoing global pandemic; - We view the following results are important additions to the science of QI programmes: - highlighting QI - specific leader characteristics as something separate to general leadership skills (p65, lines 683-4); - listed the leader’s tenacity as important for which we did not find literature (p37, line 703); - proposing a ‘life course lens’ with eight good practice p3, lines 52-3; p36, lines 672-5), in contrast to literature focusing on specific aspects of QI programmes for instance either on training or how the methodology is used, the leader, or teamwork; - the significance of a second-in-charge leader (p39, lines 740); - the importance that advisors’ technical proficiencies must be complemented with sound interpersonal skills (p37, lines 711-2; and - why it is important to address team performance in the beginning of the programme, p41, lines 787-92. |
| 11/  Results  Additionally, for GP2, you mention that they should appoint advisors with sound technical skills; who would do this? How can they ensure that have the skills required to assist the team? | We did not find literature where advisors are not described as QI experts; as far as we are concerned they are by default QI experts (references 64-6, p37, line 706). Our study points to the importance that their technical skills should be matched with their interpersonal skills, and highlights this p37, lines 711-2. We added that ascertaining their QI and interpersonal skills should be done by the agencies training QI advisors, p38, lines 714-5. |
| 12/  Results  - GP4 discusses recruiting ‘members from existing service teams known to work well together’, however, I disagree with this statement. Having a diverse team can lead to better improvements as differing ideas and approaches can lead to more successful changes, it is more about having a team that is willing to try different approaches and learn from failures. | We agree with the comment, and clarified that we do not mean recruitment from a single existing service team by adding that it could be from ‘team/s’ (p39, lines 754, 756, 757), but that the teams from which they are recruited, should have a ‘we know how to work together’ culture (p39, lines 754-8. It is our view that members coming from a ‘healthy’ existing team/s are likely to carry that culture into their QI team, and likewise may those coming from a negative culture first need induction to positive teamwork before QI activities commence. |
| 13/  Results  - GP8, I would extend this to, everyone needs to be on board in order to reap the most benefits from incorporating QI training. | Though we agree, this study focused on facility level contexts only, which we addresses with the reference to core members with influence to motivate ad hoc members + facility manager. We cannot therefore comment on buy-in from higher level management, such as district or health ministry. |
| 14/  Discussion - In the discussion you provide the implantation processes. I think it would have been easier to digest had the key points been in a table and then expanded upon in the text. | Thank you for the suggestion. Kindly see how we merged it with the text suffices, p36, line 678; p37, line 705; p38, lines 717, 726, 734; p39, line 748; p40, line 761. |
| 15/  Discussion  - A lot of the discussion, as mentioned previously, uses phrasing such as ‘this study confirms’. Although it is good to confirm others research, it is difficult to identify what is a novel contribution and what is already established knowledge. | Kindly refer to our responses to Comments 3b and 10. It is also important to hold our findings within the context of the field to which they aim to contribute. Implementation science is less concerned with the production of novelty, and more with nuancing the ‘common sense’ understandings of programming in real world settings. These nuanced understanding may otherwise be elided in the literature (for instance, in reports on impact evaluations). We stand behind the importance and contribution of our findings, and have tried to use language in this section which emphasises this (i.e., where confirmation is nonetheless novel, due to the setting or scope or nuance of our insights). |
| 16/  Minor issues: - There are minor grammatical errors throughout the paper | We did a thorough language edit and corrected the grammatical errors. |
| 17/  Minor issues: - Some of the titles are very long | We deleted subheading 2b, p23, lines 393-4, and shortened sub-heading 3, p24, line 404. |
| 18/  Minor issues: - Sometimes for the quotes it just says R instead of Researcher | We corrected it. |
| 19/  I believe this paper has some interesting concepts that can be explored further, but I really wanted to see some of the outcomes of the study in regards to the scenario it was used in and not just insights into carrying out effective QI programs. | Thank you for the suggestion. We believe that the following additions will give the reader a better “sense of the scenario in which it was implemented.”   - Types of services per facility type, added in S3 Table - Topics addressed in the change ideas: p34, lines 624-8, and S7 Table - An example of service indicators: p5-6, lines 109-10 - An example of a change idea: p33, lines 613-7 - Birth volumes reported by participants: p29, lines 518-22   Kindly also note that the purpose of the paper is to distil good practice processes and develop a framework for planning a QI programme. The main Mphatlalatsane outcome evaluation paper will address the reference “to see some of the outcomes of the study ….”. |
| 20/  I also understand the effect that COVID-19 had on this work and applaud the authors for being able to continue their research despite the difficulties. | Thank you, but the true heroes were the HCWs. It was quite humbling to engage with the healthcare workers during these trying times. |
| 21/  Currently, I do not think this paper has enough of a significant contribution to move forward and the narrative needs major adjustments. However, there is still interesting aspects of this work that could be impactful if restructured. | We trust that our responses to Comment 10 and 19 address your concern. |
| 22/  Some useful papers that I didn’t see in the citation list:   a. Implementing Evidence Based Practice nursing using the PDSA model: Process, lessons and implications, Katowa-Mukwato Patricia et al. (2021) b. Hill, J.E., Stephani, AM., Sapple, P. et al. The effectiveness of continuous quality improvement for developing professional practice and improving health care outcomes: a systematic review. Implementation Sci 15, 23 (2020). [https://doi.org/10.1186/s13012-020-0975-2](https://url.za.m.mimecastprotect.com/s/XyKVCGZXPxIL9JgVIrj9pC) c. Damschroder, L.J., Yankey, N.R., Robinson, C.H. et al. The LEAP Program: Quality Improvement Training to Address Team Readiness Gaps Identified by Implementation Science Findings. J GEN INTERN MED 36, 288–295 (2021). [https://doi.org/10.1007/s11606-020-06133-1](https://url.za.m.mimecastprotect.com/s/bJ32CJZKVAIQo8X7fKCYpn) | Thanks for the resources which we used as follow:   a. p37, lines 695-7, ref 58. b. p4, lines 78-9, ref 13. c. p38, lines 729-30, ref 69. |
